# Lower Fiber Consumption in Women with Polycystic Ovary Syndrome: A Meta-Analysis of Observational Studies

**DOI:** 10.3390/nu14245285

**Published:** 2022-12-12

**Authors:** Wing Ting Leung, Zhijing Tang, Yuanyuan Feng, Haiyun Guan, Zengshu Huang, Wei Zhang

**Affiliations:** 1 Department of Reproductive Endocrinology, Obstetrics and Gynecology Hospital, Fudan University, Shanghai 200011, China; 2 Shanghai Key Laboratory of Female Reproductive Endocrine Related Diseases, Shanghai 200011, China

**Keywords:** polycystic ovarian syndrome (PCOS), dietary fiber, observational studies, meta-analysis

## Abstract

Polycystic ovary syndrome is a common endocrine disorder associated with metabolic abnormalities and gut microbiota dysbiosis. The deficiency of dietary fiber, a crucial nutrient in the daily diet, is also associated with a wide range of metabolic and reproductive abnormalities, as well as an altered gut microbial ecosystem. This study is a meta-analysis to summarize the available evidence on the dietary fiber intake level in PCOS patients. Databases of PubMed, Embase, Cochrane Library, Web of Science, and ClinicalTrials.gov were searched for observational studies, and 13 studies were finally included. The pooled standardized mean difference (SMD) with the 95% confidence interval (CI) of daily dietary fiber intake and total energy intake were calculated using the random-effects model. The pooled result (12 studies) on absolute dietary fiber intake showed that while there was no significant difference in the total energy intake [−0.17 (−0.44, 0.09), *p* = 0.208], the dietary fiber intake was significantly lower in PCOS women than those of controls [−0.32 (−0.50, −0.14), *p* < 0.001]. However, significant heterogeneity was detected across the studies (*I*^2^ = 65.6%, *p* = 0.001). Meta-regression suggested that geographic region and dietary assessment method may confer borderline significance of influence on the heterogeneity. The pooled result (two studies) on dietary fiber intake which adjusted for total energy intake, however, showed no significant difference [−2.11 (−4.77, 0.56), *p* = 0.122]. In subgroup analyses based on absolute dietary fiber intake, a lower dietary fiber intake in PCOS was observed in studies conducted in Asia, adopted food diary or records or food recall as the dietary assessment method, had a case–control study design, or used Rotterdam criteria for PCOS diagnosis. The difference in SMD was still significant in the adult subgroup or in studies matched or unmatched for age.

## 1. Introduction

Polycystic ovary syndrome (PCOS) is a heterogeneous disorder in women of reproductive age characterized by a combination of signs and symptoms, including hyperandrogenism, ovulatory dysfunction, and polycystic ovary morphology [1]. Although not part of the diagnostic criteria, metabolic abnormalities, including insulin resistance, obesity, and dyslipidemia, often coexist in PCOS [2,3,4]. Affecting 6–20% of women worldwide, PCOS is by far the most prevalent endocrinopathy of females [5,6]. However, the pathogenesis remains largely unknown. Recent studies have addressed the critical role of gut microbial dysbiosis in the development of PCOS. It is now acknowledged that PCOS is a multifactorial disorder with strong internal (e.g., gut microbiota) and external (e.g., lifestyle factors) environmental influences [2,7]. Lifestyle, mainly dietary, management is recommended as the first-line therapy for PCOS [8,9,10]. However, due to the lack of reliable evidence, currently, there are no specific suggestions for dietary intervention in PCOS [11].

Dietary fiber, a crucial dietary component which is found high amounts in fruit, vegetables, and whole grains, has been identified to have multiple beneficial effects in humans, including regulations in weight control, inflammation, insulin resistance, lipid metabolism, and hormonal derangements [12,13,14,15]. Adequate intakes of dietary fiber can confer benefits to protection against type 2 diabetes, cardiovascular diseases, and even malignancies such as colorectal and breast cancer [16,17,18]. The benefits of dietary fiber on health were not confined to specific fiber types and were apparent across the range of intakes [12]. Moreover, reliable studies have also revealed the effects of dietary fiber in shaping gut microbial compositions and modulating the microbial metabolites which are considered important for host health [19,20,21]. On the contrary, inadequate intake of dietary fiber is associated with a wide range of detrimental effects. For example, studies have shown that lower fiber intake is associated with a higher insulin level [22], enhanced inflammatory response [23], and higher risks for diseases such as diabetes, cardiovascular diseases, and colon cancers [24,25]. Moreover, a diet low in dietary fiber is also detrimental to the maintenance of diverse microbiota and the production of key metabolites, such as short-chain fatty acids (SCFAs), resulting in adverse effects on host health [26,27]. 

Considering the benefits of dietary fiber on human metabolism and gut microbiota which are also often found abnormal in PCOS, it is reasonable to question: Do women with PCOS consume enough dietary fiber? Or rather, is there a difference between PCOS and non-PCOS women in daily dietary fiber intake? Studies so far have provided inconsistent results. Therefore, we performed this meta-analysis to investigate the dietary fiber intake level in PCOS women. The results may provide clues for PCOS pathogenesis from the dietary perspective, and may provide evidence for the development of dietary interventions for PCOS treatment. 

## 2. Materials and Methods

The meta-analysis was conducted in accordance with the Meta-Analysis of Observational Studies in Epidemiology (MOOSE) [28] guideline and the Preferred Reporting Items for Systematic Reviews and Meta-Analyses (PRISMA) guideline [29].

### 2.1. Data Sources and Searches Strategy

A comprehensive literature search in the PubMed, Embase, Cochrane Library, Web of Science, and ClinicalTrials.gov online databases in December 2021 was conducted to identify all the available studies published. The following search terms or text terms were used: (“polycystic ovary syndrome” OR “polycystic ovar*” OR “stein leventhal” OR “PCOS” OR “PCO”) AND (“dietary fiber” OR “fiber*” OR “fibre*”). To identify any other eligible studies that were not identified by our search strategy, we also manually reviewed the reference lists of identified papers. No language or region restrictions were applied. Unpublished studies were not included in this meta-analysis.

### 2.2. Study Selection

The study selection was performed independently by two investigators (W.T.L. and Z.T.). Duplicate studies were screened and removed firstly by reference manager software (EndNote version 20.2; Thomson Reuters Corp., New York, NY, USA) and later by manual check. Then, titles and abstracts were screened for relevance. Records that were deemed irrelevant were excluded. For studies that were uncertain of eligibility, full texts were reviewed. Studies were included if they met all of the inclusion criteria: (1) had an observational design (e.g., cohort, case–control, or cross-sectional); (2) investigated the dietary fiber intake level in women with PCOS and women controls; (3) reported the means and SDs of daily dietary fiber intake data in both groups, or provided data for their calculation. Studies were excluded if they met any of the exclusion criteria: (1) duplicated publications; (2) non-original articles (e.g., review, meta-analysis, or conference abstract); (3) not conducted in human subjects; (4) lack a non-PCOS control group; (5) PCOS cases were self-reported without further confirmation of diagnosis; (6) dietary fiber intake not adjusted for total energy intake, or data of total energy intake not given; (7) incomplete data. When multiple studies reported data based on overlapping populations, the one with more informative data was considered. Any disagreements regarding study eligibility from the authors were discussed. If the disagreement remained, further discussion with a third author was performed until a consensus was reached.

### 2.3. Data Extraction

The following information was extracted from each included study onto a standardized form: first authors’ names, year of publication, study design, country where the study was conducted, period of enrollment for case–control and cross-sectional studies, or follow-up for cohort studies, criteria for PCOS definition, method of dietary fiber intake assessment, whether data were adjusted for total energy intake, sample sizes of cases and controls, means and standard deviations of daily fiber intake and total energy intake, subject age and BMI, and matched or adjusted confounders. If the included studies reported dietary fiber intake data stratified by BMI classification, the above information of each weight group was also separately recorded. If only medians and IQRs of daily fiber intake were reported, formulas proposed by Wan et al. [30] were used to calculate means and SD values. If the standard error of mean (SEM) of daily dietary fiber was given, the SD was calculated by the formula: $SEM=SD/\sqrt{n}$. The process of data extraction was also conducted by the two authors independently. Any disagreements between the two authors were discussed by referring back to the original text in case of incorrect or unclear data.

### 2.4. Quality Assessment

Two investigators (W.T.L. and Z.T.) conducted the quality assessment of the included studies using the Newcastle–Ottawa scale (NOS) [31,32], as recommended by the Cochrane Collaboration. The scaling used three parameters for quality assessment in case–control or cohort studies: selection (maximum score = 4), comparability (maximum score = 2), and exposure/outcome (for case–control or cohort studies, respectively; maximum score = 3). A maximum of 9 points can be allocated to each study. Studies scoring ≥6 points were considered of high quality.

### 2.5. Statistical Analysis 

All statistical analyses were performed using STATA version 15.1 (Stata Corporation, College Station, TX, USA). The standard mean difference (SMD) and 95% confidence intervals (CI) of daily dietary fiber intake and total energy intake were calculated. Statistical heterogeneity of pooled results was assessed by the chi-square (χ^2^) test and quantified by the I-square *(I^2^*) statistic, which represents the proportion of total variation explained by variation among studies. Heterogeneity was considered significant if *p* < 0.1 or *I*^2^ > 50% [33,34]. The random-effects or fixed-effects model [33,35] was applied to calculate the SMD and 95% CIs according to the result of the heterogeneity test.

Our primary result compared the daily dietary fiber intake between PCOS women and non-PCOS. Since the dietary fiber intake in most of the studies was not adjusted for total energy intake, we also pooled and compared the overall energy intake given by these studies to yield more reliable conclusions. In order to evaluate the potential reasons for heterogeneity, subgroup analysis according to factors that may contribute to the heterogeneity was performed. These factors include: geographic locations (continents), dietary assessment methods, study designs, criteria for PCOS definition, and adjustment for BMI and age. Meta-regression was conducted to evaluate the heterogeneity brought about by the potential covariates. Influence analysis was performed by omitting one study at a time to assess the influence of each study on the overall estimate. Publication bias was indicated by the visualization of funnel plots and evaluated by Begg’s test and Egger’s tests [36,37]. All reported probabilities (*p* values) were two-sided, with *p* < 0.05 considered to be statistically significant.

## 3. Results

### 3.1. General Characteristics of Included Studies

**Figure 1:**
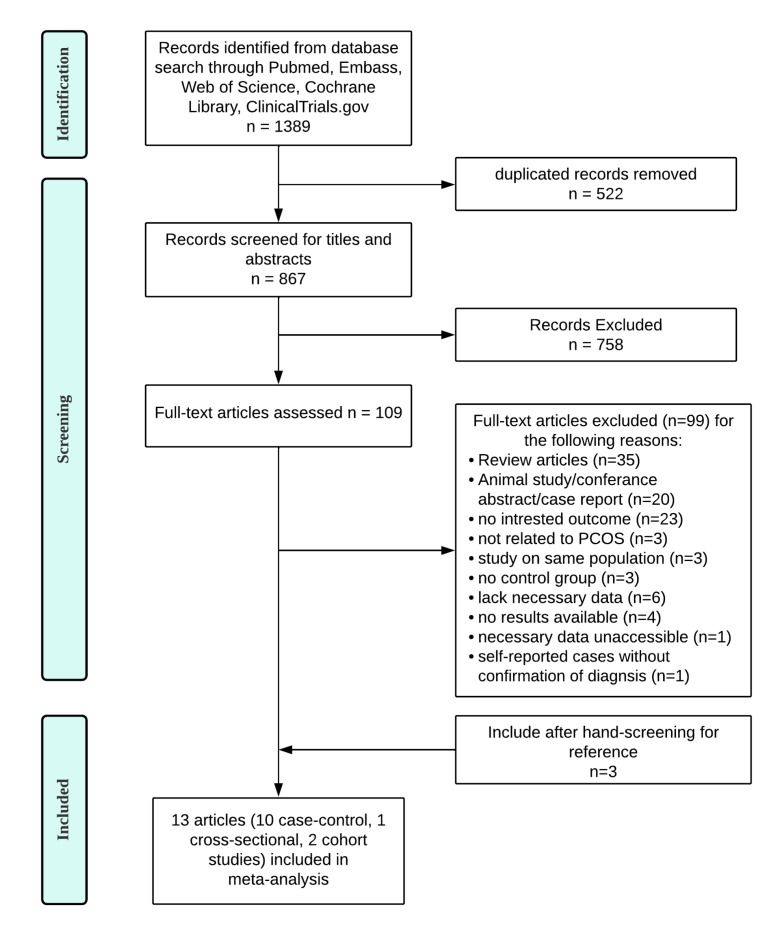
Flow diagram of the literature search.

Through a comprehensive search, a total of 1389 articles were identified. After the selection process, 13 articles, including 10 case–control studies [38,39,40,41,42,43,44,45,46,47], 1 cross-sectional study [48], and 2 cohort studies [49,50] were ultimately deemed eligible and included in the meta-analysis. A flow chart of detailed steps of the literature search and selection process is presented in Figure 1. 

The included articles were all published in English and published between 2006 and 2021. The studies cumulatively reported data on a total of 2469 participants, including 1130 PCOS cases and 1339 controls. Among the 13 studies, five were conducted in Europe (Italy, Spain, Turkey, Poland), four from Asia (China, Iran), three from North America (USA, Canada), and one from South America (Brazil). Two studies [38,39] enrolled only overweight or obese women as participants, while the other studies did not limit the BMI for inclusion. Two studies [40,42] reported results stratified by the weight ranges of PCOS cases and controls, and we considered each weight class as a separate data. With regard to the data adjustment, one study [49] provided both adjusted and unadjusted data for total energy intake, one study [41] only presented adjusted data, and the rest of the studies [38,39,40,42,43,44,45,46,47,48,50] reported only unadjusted data. Table 1 summarizes the characteristics of each included study.

According to the NOS system, 9 out of 13 studies were considered high quality, with three studies scoring 5 points and one study scoring 4 points. Table S1 presents the detailed scoring and total score for the included studies. 

### 3.2. Daily Dietary Fiber Intake in PCOS and Controls

**Figure 2:**
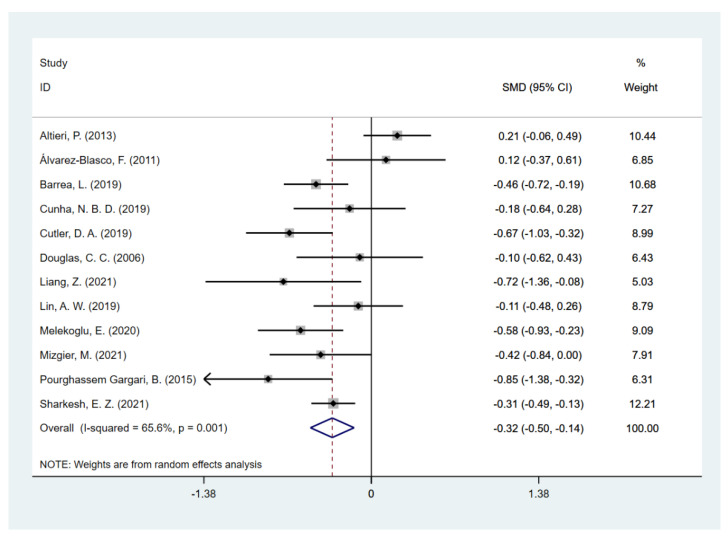
Meta-analysis of daily dietary fiber intake on studies unadjusted for total energy intake [38,39,40,40,42,43,44,45,46,47,48,49,50].

**Figure 3:**
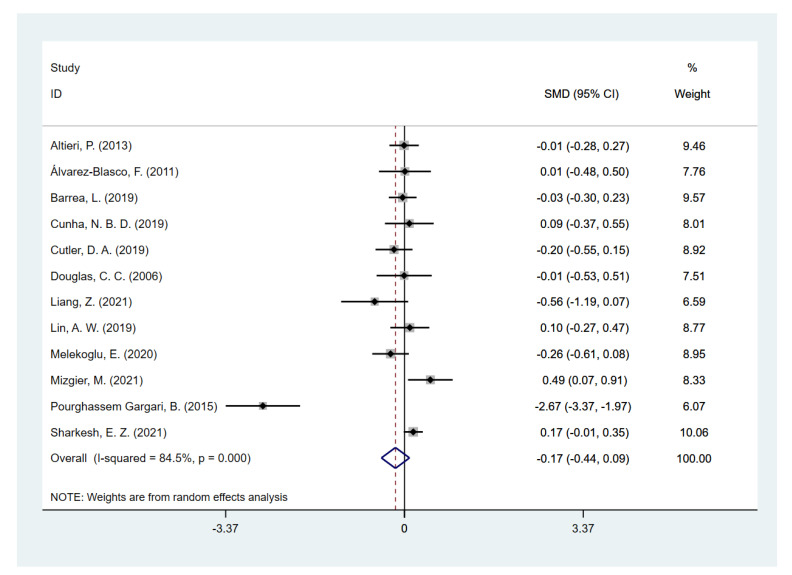
Meta-analysis of daily total energy intake on studies unadjusted for total energy intake [38,39,40,42,43,44,45,46,47,48,49,50].

**Figure 4:**
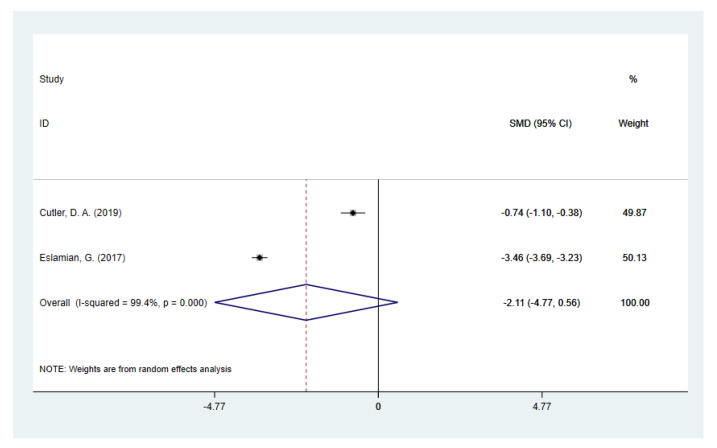
Meta-analysis of daily dietary fiber intake on studies adjusted for total energy intake [41,49].

Since one included study [49] provided both types of data that adjusted or unadjusted for total energy intake, and the rest of the studies provided either unadjusted (12 studies) or adjusted (one study) data, the two types of data were separately pooled in the meta-analysis to compare the dietary fiber intake in PCOS women and controls. Pooling of unadjusted data given by 12 studies revealed that the daily dietary fiber intake level was significantly lower in PCOS women [SMD (95% CI): −0.32 (−0.50, −0.14), *p* for *Z* < 0.001; *I*^2^ = 65.6%, *p* for *I*^2^ = 0.001] compared to the non-PCOS controls (Figure 2), while there was no significant difference in total energy intake [SMD (95% CI): −0.17 (−0.44, 0.09), *p* for *Z* = 0.208; *I*^2^ = 84.5%, *p* for *I*^2^ < 0.001; Figure 3]. The two studies [41,49] that provided adjusted data both reported a significantly lower fiber intake level in PCOS. However, the pooled results did not show statistical significance [SMD (95% CI): −2.11 (−4.77, 0.56), *p* for *Z* = 0.122; *I*^2^ = 99.4%, *p* for *I*^2^ < 0.001; Figure 4]. Since substantial heterogeneity was observed across the studies, the random-effects model was used for analyses. 

We also extracted and pooled the data according to BMI classification (overweight/obese or lean) from the included studies that stratified fiber intake by BMI range. As shown in Supplementary Figure S1, the overall effect sizes showed no significant difference [SMD (95% CI): −0.15 (−0.60, 0.31), *p* for *Z* = 0.531; *I*^2^ = 64.7%, *p* for *I*^2^ = 0.037] in dietary fiber intake between overweight or obese PCOS women and controls. Moreover, no significant difference was found between lean PCOS women and controls [SMD (95% CI): −0.03 (−0.82, 0.75), *p* for Z = 0.938; I^2^ = 52.8%, *p* for I^2^ = 0.145]. Meanwhile, no significant difference was found in total energy intake in both comparisons [SMD (95% CI): −0.04 (−0.26, 0.18), *p* for *Z* = 0.748; and −0.04 (−0.56, 0.48), *p* for *Z* = 0.872, respectively; Supplementary Figure S2].

### 3.3. Subgroup Analysis

There were only two studies that reported the daily dietary fiber intake data adjusted for total energy, which is too few for further subgrouping. Studies that provided the unadjusted data (a total of 12 studies) were included in the subgroup analyses. Table 2 shows the comparisons of daily fiber intake between PCOS and controls in the pre-planned subgroup meta-analyses.

Since the dietary pattern could vary greatly with different geographic locations, subgroup analysis stratified by geographic location (continent) was conducted. Significant results were observed in the studies conducted in Asia [SMD (95% CI): −0.53 (−0.78, −0.27), *p* for *Z* < 0.001], but not found in Europe, North America, or South America. When we stratified the study by the dietary assessment method, studies that used the food diary or records [SMD (95% CI): −0.32 (−0.58, −0.05), *p* for *Z* = 0.019], and studies that used food recall [SMD (95% CI): −0.73 (−1.07, −0.39), *p* for *Z* < 0.001], but not FFQ, reported significantly lower fiber intake in PCOS women. In stratified analysis by the study design, a significant association was found for case–control studies [SMD (95% CI): −0.28 (−0.50, −0.06), *p* for *Z* = 0.012], but not for cohort studies [SMD (95% CI): −0.42 (−0.98, 0.15), *p* for *Z* = 0.147]. Only one included study [48] was cross-sectional and it reported a significantly lower level of dietary fiber intake in PCOS. When we pooled the results in adults other than in adolescent girls, the difference in dietary fiber intake was still significant [SMD (95% CI): −0.31 (−0.51, −0.12), *p* for *Z* = 0.002]. For the analysis by criteria for PCOS definition, studies that used the Rotterdam criteria for PCOS diagnosis reported significantly lower fiber intake in PCOS women [SMD (95% CI): −0.37 (−0.57, −0.18), *p* for *Z* < 0.001]. The results were also significant in studies matched or not matched by age [SMD (95% CI): −0.25 (−0.50, −0.00), *p* for *Z* = 0.047; −0.44 (−0.72, −0.16), *p* for *Z* = 0.002], but not for studies matched for BMI [SMD (95% CI): −0.31 (−0.65, 0.03), *p* for *Z* = 0.071]. No difference was found in total energy intake between PCOS and controls in each subgroup mentioned above.

### 3.4. Meta-Regression

As shown in Figure 2, high between-study heterogeneity (*I*^2^ = 65.6%) was demonstrated. To further investigate the contribution of available covariates on the high heterogeneity demonstrated in the above studies, univariate meta-regression with the covariates of continents, age group, study design, individual age match, individual BMI match, PCOS definition, dietary assessment method, publication year and country were conducted, respectively. The *p* values from the meta-regression of the above covariates are listed in Table 3. The results show that these covariates suggested did not confer a significant influence on the between-study heterogeneity. Only borderline significance of influence was noted in the geographic region and dietary assessment method.

### 3.5. Influence Analysis and Publication Bias

Influence analysis showed that the pooled result of the association between dietary fiber intake and PCOS was not significantly influenced by a single study (Supplementary Figure S3). Through visual observation, the distribution of all studies on funnel plots appeared to be symmetrical, suggesting no obvious publication bias existed (Supplementary Figure S4). Consistently, Egger’s test and Begg’s test revealed no evidence of publication bias (*p* = 0.434 and *p* = 0.784, respectively, Supplementary Figures S5 and S6). 

## 4. Discussion

To our knowledge, this is the first meta-analysis that investigated the dietary fiber intake in PCOS, and also the first meta-analysis to compare the consumption of specific dietary components in women with and without PCOS. 

On pooling the 12 studies, which provided an absolute value of fiber intake, we confirmed that, while there was no significant difference in the total energy intake, PCOS women consumed a significantly lower level of dietary fiber compared with the non-PCOS controls. Influence analysis further confirmed the validity and robustness of the main result. Subgroup analyses were conducted for further interpretation. In the subgroup analysis using geographic location, the difference in dietary fiber intake was found significant in Asia with acceptable between-study heterogeneity, but not found in the other continents. Lower fiber intake was also found in studies that used food diary/records or food recall as the dietary assessment method. In addition, studies that had a case–control design or cross-sectional design, or studies that acquired the Rotterdam criteria for PCOS definition, also showed a significant difference. When the study on adolescent PCOS was not included, the result was still significant. In the subgroup analysis by whether studies were adjusted or matched by age, we did not find a meaningful influence on the main results. 

On the other hand, however, no significant difference was found when pooling the two studies that adjusted fiber intake by total fiber intake, although they both reported a significantly lower fiber intake in PCOS women compared with the controls. The high heterogeneity between the study may explain the inconsistency with the main result. In addition, when we pooled the data stratified by BMI and subgroup according to BMI classification, no difference was found in the dietary fiber intake or in total energy intake.

A few studies have corroborated an association between inadequate dietary fiber intake and metabolic disturbance in PCOS. For instance, research has reported an inverse correlation between dietary fiber intake and body fat accumulation, insulin resistance, fasting insulin, and glucose tolerance in PCOS women [40,49]. A recent randomized controlled trial [51], which used the resistant dextrin (a soluble dietary fiber) as an intervention in PCOS women, showed significant improvements in metabolic parameters and inflammatory markers, including a decrease in the serum level of LDL-C, triglycerides, total cholesterol, and high-sensitivity C-reactive protein. Moreover, research has also revealed a possible beneficial effect of fiber intake on hormonal regulation in PCOS. A study [48] from Italy, which investigated 224 women with and without PCOS, reported that, following adjustments for BMI and total energy intake, the testosterone level in PCOS women was significantly negatively correlated with adherence to a Mediterranean diet (a dietary pattern rich in fiber) or fiber consumption. The clinical trial by Gholizadeh Shamasbi [51] also reported an improvement in hyperandrogenism and hirsutism as well as menstrual cycle irregularity in PCOS following dietary fiber intervention. Similar results were also found in another study [49]. 

Since lower dietary fiber intake is indicated to be associated with the metabolic and hormonal disturbances in PCOS, and our result has confirmed a significantly lower level of dietary fiber intake in PCOS women compared with controls, it brings up an interesting question on how dietary fiber intake may influence PCOS. One of the most important physiological roles of dietary fiber in humans is that through direct interaction with gut microbes, it can beneficially shape the microbial ecosystem and enhance the production of key microbial metabolites [52,53,54]. On the contrary, low dietary fiber intake not only leads to progressive loss of microbial diversity [21,55], but also shifts the microbial metabolism towards utilization of less favorable substrates [56,57] and degradation of protective mucin [58], which are detrimental to the hosts. In PCOS, numerous studies have demonstrated a significant decrease in biodiversity in the gut microbiome [59,60,61,62,63]. Whether a low dietary fiber intake contributes to the variation in microbial communities remains unclear. 

Another mechanism by which dietary fiber intake may affect PCOS is the modulation of microbial metabolites. Short-chain fatty acids (SCFAs), which are key microbial metabolites produced in the colon through fermentation of dietary fiber by gut microbes [54,64], are famous for possessing functional roles in regulating host metabolism [53,65,66,67], immune system [66,68,69], and cell proliferation [70,71]. A decrease in fiber intake could possibly affect the production of metabolites, especially SCFAs, and finally influence overall health and well-being. Considering that PCOS women consume less dietary fiber, whether there is a reduction in SCFA production remains unclear. Furthermore, whether increasing SCFA level by modulating dietary fiber intake or through dietary supplementation has a beneficial effect on PCOS warrants further investigation.

Several limitations of the present meta-analysis should be considered. First, the significant heterogeneity detected could not be sufficiently explained by further meta-regression or subgroup analyses. We attributed the heterogeneity to a number of factors, which include: the severity or subtypes of PCOS, inconsistent exclusion criteria, inconsistent nutrient analysis method, and measurement or reporting inaccuracy of diet. Second, there were a limited number of studies included in certain subgroups, such as the cohort study or adolescent subgroup, making the results lack certain representation. Third, most of the controls in studies were enrolled from outpatient visitors and may have resulted in a lack of representativeness. Fourth, energy adjustment is advantageous in analyses of diet–disease associations since it mitigates the influence of body size, metabolic efficiency, physical activity, etc., and also diminishes measurement errors [72,73]. However, only two included studies [41,49] conducted energy adjustment. Thus, it was difficult to yield stronger evidence appreciably for result interpretations. In order to diminish the influence of total energy intake and give more comprehensive interpretations of the results, we also pooled and compared the overall energy intake. Fifth, as only four studies [38,39,40,42] presented information based on BMI classification, it was difficult to confirm the dietary fiber intake level in overweight or obese PCOS women, who are the focus group for dietary or lifestyle interventions. How dietary fiber intake differs in this group remains to be evaluated in future investigations.

## 5. Conclusions

The present meta-analysis showed that the dietary fiber intake level may be significantly lower in women with PCOS, although there was a high heterogeneity of included studies. Decreased dietary fiber intake might play a role in the development of PCOS and warrant attention when considering the dietary intervention strategy for this clinical population. In the future, more studies are needed to further confirm our observations, and to investigate whether and how an increase in dietary fiber intake can be beneficial as a dietary approach to improve PCOS health outcomes.

## Figures and Tables

**Table 1:** Characteristics of the included studies.

First Author, (Reference), Year, Country	Study Design (Period of Enrollment)	PCOS Definition	Dietary Assessment Method	Adjusted for Total Energy	Group	n	Mean Daily Fiber Intake (g/d)	SD (g/d)	*p* Value	Total Energy Intake * (kcal/d)	Age* (Year)	BMI* (kg/m^2^)	Matched/Adjusted Factors
Altieri P [38], 2013, Italy	Case–control (2005–2010)	Rotterdam	7-day food diary	No	overweight/obese PCOS	100	19.30	5.00	0.025	2220.00 ± 457.00	27.7 ± 5.2	34.7 ± 5.5	Age, BMI
					overweight/obese controls	100	18.20	5.30		2223.00 ± 405.00	28.4 ± 5.8	34.8 ± 5.4	
Álvarez-Blasco F [39], 2011, Spain	Case–control (2002–2005)	AES	FFQ/ not stated	No	overweight/obese PCOS	22	23.00	11.00	0.361	2374.00 ± 681.00	26.3 ±7.6	35.2 ± 6.7	Age
					overweight/obese controls	59	22.00	7.00		2368.00 ± 702.00	32.2 ± 7.5	34.8 ± 6.1	
Barrea L [48], 2019, Italy	Cross-sectional (2014–2019)	Rotterdam	7-day food record	No	PCOS	112	15.43	3.66	0.001	2245.31 ± 290.75	24.21 ± 5.47	30.95 ± 5.66	Age, BMI
					controls	112	17.22	4.19		2254.84 ± 272.37	24.07 ± 5.05	30.76 ± 5.60	
Cunha NBD, [40], 2019, Brazil	Case–control (2015–2017)	Rotterdam	7-day food report	No	all PCOS	39	11.50	5.38	0.580	1651.42 (1184.19–1949.22)	25.17 ± 3.86	24.43 (20.90–33.84)	Age, BMI
					all controls	34	12.65	7.46		1487.88 (1240.79–1903.91)	25.67 ± 4.42	23.95 (21.62–31.01)	
					lean PCOS	20	15.31	11.29	NA	1683.64 (1415.49–2156.04)	(18–35)	NA	
					lean controls	19	12.48	5.96		1704.98 (1120.49–2120.01)		NA	
					overweight/obese PCOS	19	9.50	3.18	NA	1479.12 (1030.53–1922.42)		NA	
					overweight/obese controls	15	12.87	8.68		1372.38 (1258.75–1665.95)		NA	
Cutler DA [49], 2019, Canada	Cohort (2014–2016)	Rotterdam	3-day food record	No	PCOS	87	19.80	6.03	≤ 0.01	1783.00 (1516.00–1966.00)	30.7 ± 4.6	29.0 ± 7.1	Unmatch
				Yes	PCOS	87	19.77	6.26					
				No	controls	50	24.83	9.46		1815.00 (1578.00–2083.00)	35.7 ± 5.2	24.1 ± 5.1	
				Yes	controls	50	25.03	8.40					
Douglas CC [50], 2006, USA	Cohort (not specified)	NIH (1990)	4-day food record	No	PCOS	30	14.90	3.30	0.761	1781.50 ± 444.80	28.9 ± 6.3	29.7 ± 4.8	Age, race, BMI
					controls ^a^	27	15.40	6.80		1783.90 ± 379.30	28.9 ± 6.5	29.1 ± 4.8	
Eslamian G [41], 2017, Iran	Case–control (2012–2014)	Rotterdam	FFQ/stated	Yes	PCOS	281	12.00	5.30	≤ 0.001	3215.00 ± 721.00	28.8 ± 7.6	31.2 ± 7.5	age
					controls	472	29.50	4.90		2489.00 ± 561.00	29.4 ± 7.5	25.9 ± 3.8	
Liang Z [42], 2021, China	Case–control (not stated)	Rotterdam	24-h food recall	No	all PCOS	20	8.99	2.10	<0.05	1578.75 ± 334.98	26.54 ± 5.17	23.90 ± 4.41	Age, BMI
					all controls	20	11.43	4.28		1780.00 ± 379.44	27.60 ± 5.06	23.24 ± 3.69	
					lean PCOS	10	9.04	2.43	NS	1568.80 ± 351.01	24.13 ± 2.45	20.46 ± 1.58	
					lean controls	10	10.78	4.27		1728.50 ± 417.00	25.08 ± 3.59	20.43 ± 1.19	
					overweight/obese PCOS	10	8.94	1.84	<0.05	1588.70 ± 336.84	28.94 ± 6.13	27.34 ± 3.51	
					overweight/obese controls	10	12.08	4.42		1831.50 ± 352.37	30.12 ± 5.20	26.05 ± 3.13	
Lin AW, [43] 2019, USA	Case–control (2013–2018)	Rotterdam	FFQ	No	PCOS	80	24.00	8.99	0.49	2218.00 (2017.00–2419.00)	26.8 (25.4–28.1)	31.5 (29.5–33.4)	Unmatch
					controls	44	25.00	9.87		2180.00 (1866.00–2494.00)	29.5 (27.5–31.4)	28.0 (26.1–29.8)	
Melekoglu E [44], 2020, Turkey	Case–control (2013–2013)	Rotterdam	3-day food record	No	PCOS	65	20.70	7.70	<0.001	1732.70 ± 474.00	26.45 ± 7.42	29.7 ± 9.13	age
					controls	65	25.80	9.70		1854.40 ± 452.80	26.52 ± 8.90	22.6 ± 6.60	
Mizgier M [45], 2021, Poland	Case–control (not stated)	Rotterdam	3-day food record	No	PCOS	61	15.53	6.91	0.069	1663.50 (1444.70–1788.40)	16 (15–17)	NA	Age
					controls	35	18.27	5.93		1474.01 (1189.44–1746.39)	15 (15–17)	NA	
Pourghassem Gargari B [46], 2015, Iran	Case–control (2009–2010)	Rotterdam	3-day food recall and FFQ	No	PCOS	30	6.00	1.00	NS	1334.90 ± 143.40	25.83 ± 4.00	25.00 ± 3.61	BMI
					controls	30	6.70	0.60		1716.10 ± 142.07	26.06 ± 4.44	23.68 ± 3.07	
Sharkesh EZ [47], 2021, Iran	Case–control (2019–2020)	Rotterdam	FFQ	No	PCOS	203	38.01	18.21	<0.001	2500.07 ± 696.19	28.98 ± 5.43	25.74 ±5.44	Unmatch
					controls	291	44.73	23.47		2388.03 ± 657.88	30.15 ± 6.21	23.65 ±3.90	

Abbreviations: n number of participants; SD, standard deviation; FFQ, Food Frequency Questionnaire; BMI, body mass index, NS, no significant; NA, not available; * Values are presented as mean ± SD, or median (interquartile range), or (range); ^a^ Includes underweight participants and those who did not state their weight.

**Table 2:** Subgroup analysis of dietary fiber intake and PCOS.

Subgroup	N	SMD (95% CI)	Test of SMD = 0		Heterogeneity		Articles Included
			*Z*	*p* for *Z*	*I*^2^ (%)	*p* for *I*^2^	
Geographic location							
Asia	4	−0.53 (−0.78, −0.27)	4.03	**<0.001**	46	0.135	[42,44,46,47]
North America	3	−0.31 (−0.71, 0.09)	1.52	0.128	65.2	0.056	[43,49,50]
Europe	4	−0.14 (−0.52, 0.24)	0.72	0.470	79.1	0.002	[38,39,45,48]
South America	1	−0.18 (0.64, 0.28)	0.76	0.447	-	-	[40]
Dietary assessment							
Food diary/records	7	−0.32 (−0.58, −0.05)	2.35	**0.019**	73.1	0.001	[38,40,44,45,48,49,50]
FFQ	3	−0.18 (−0.41, 0.05)	1.51	0.131	37.7	0.201	[39,43,47]
Food recall	2	−0.73 (−1.07, −0.39)	3.83	**<0.001**	0.0	0.768	[42,46]
Study design							
Case–control	9	−0.28 (−0.50, −0.06)	2.51	**0.012**	68.1	0.001	[38,39,40,42,43,44,45,46,47]
Cohort	2	−0.42 (−0.98, 0.15)	1.45	0.147	69.1	0.072	[49,50]
Cross-sectional	1	−0.46 (−0.72, −0.19)	3.36	**0.001**	-	-	[48]
Adult or Adolescent							
Adult	11	−0.31 (−0.51, −0.12)	3.17	**0.002**	68.5	0.000	[38,39,40,42,43,44,46,47,48,49,50]
Adolescent	1	−0.42 (−0.84, 0.00)	1.95	0.052	-	-	[45]
PCOS definition							
Rotterdam	10	−0.37 (−0.57, −0.18)	3.77	**<0.001**	68.1	0.001	[38,40,42,43,44,45,46,47,48,49]
AES	1	0.12 (−0.37, 0.61)	0.48	0.628	-	-	[39]
NIH	1	−0.10 (−0.62, 0.43)	0.36	0.720	-	-	[50]
Adjustment or match for confounders							
age							
Yes	7	−0.25 (−0.50, −0.00)	1.99	**0.047**	67.8	0.003	[38,40,42,44,45,48,50]
No	5	−0.44 (−0.72, −0.16)	3.10	**0.002**	63.7	0.041	[39,43,46,47,49]
BMI							
Yes	6	−0.31 (−0.65, 0.03)	1.81	0.071	75.5	0.001	[38,40,42,46,48,50]
No	6	−0.35 (−0.55, −0.15)	3.43	**0.001**	51.6	0.066	[39,43,44,45,47,49]

Bold indicates a significant difference in the subgroup.

**Table 3:** Meta-regression of covariates possible for heterogeneity.

Covariates for Meta-Regression	*p* Values
Continent (Asia, North America, Europe, South America)	0.060
Age group (adult, adolescent)	0.777
Study design (case–control, cross-sectional, cohort)	0.498
Individual age match (yes, no)	0.317
Individual BMI match (yes, no)	0.817
PCOS definition (Rotterdam, AES, physician-confirmed but criteria not stated)	0.234
Dietary assessment method (FFQ, food diary/record, food recall)	0.058
Publication year (2000s, 2010s, 2020s)	0.221
Country (Italy, Spain, Brazil, Canada, USA, Iran, China, Turkey, Poland, Australia)	0.061

## Data Availability

All the data are available in the manuscript.

## Supplementary Materials

The following supporting information can be downloaded at: https://www.mdpi.com/article/10.3390/nu14245285/s1, Figure S1: Meta-analysis of dietary fiber intake by BMI classification; Figure S2: Meta-analysis of total energy intake by BMI classification; Figure S3: Influence analysis assessing the sensitivity of the pooling result on every single study; Figure S4: Funnel plot of the meta-analysis comparing dietary fiber intake on studies unadjusted for total energy intake; Figure S5: Egger’s publication bias plot; Figure S6: Begg’s publication bias plot; Table S1: Newcastle-Ottawa quality assessment of the included studies.
