# Supplementary material for: Lower Fiber Consumption in Women with Polycystic Ovary Syndrome: A Meta-Analysis of Observational Studies
Source: Nutrients. 2022 Dec 12;14(24):5285. doi: 10.3390/nu14245285 (PMC9785338; doi:10.3390/nu14245285)
Supplement: Supplementary file 1 [file nutrients-14-05285-s001.zip › nutrients-2018969-supplementary materials.pdf]

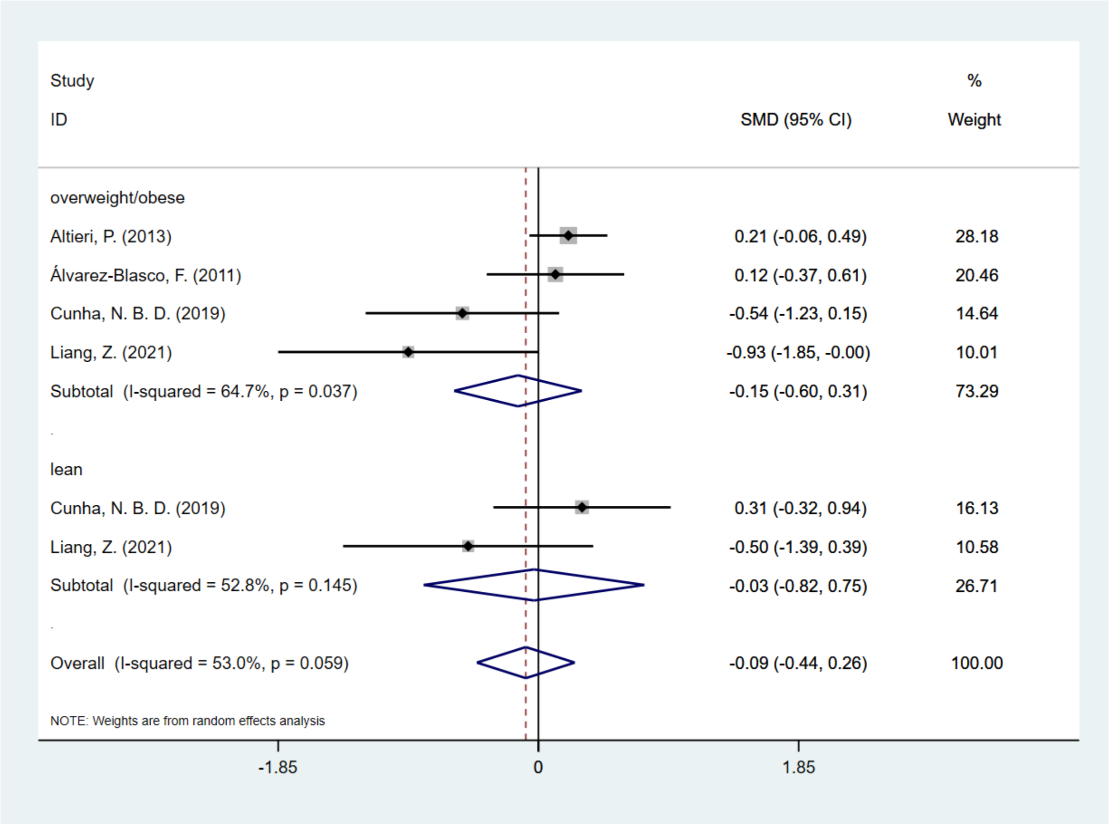

Figure S1. Meta-analysis of dietary fiber intake by BMI classification [38–40,42]. BMI is classified in accordance with WHO guideline (overweight/obese defined as BMI≥25 kg/m^2) for studies conducted outside of Asia, and in accordance with Asia-Pacific guideline (overweight/obese defined as BMI≥23 kg/m^2) for study conducted in Asia.

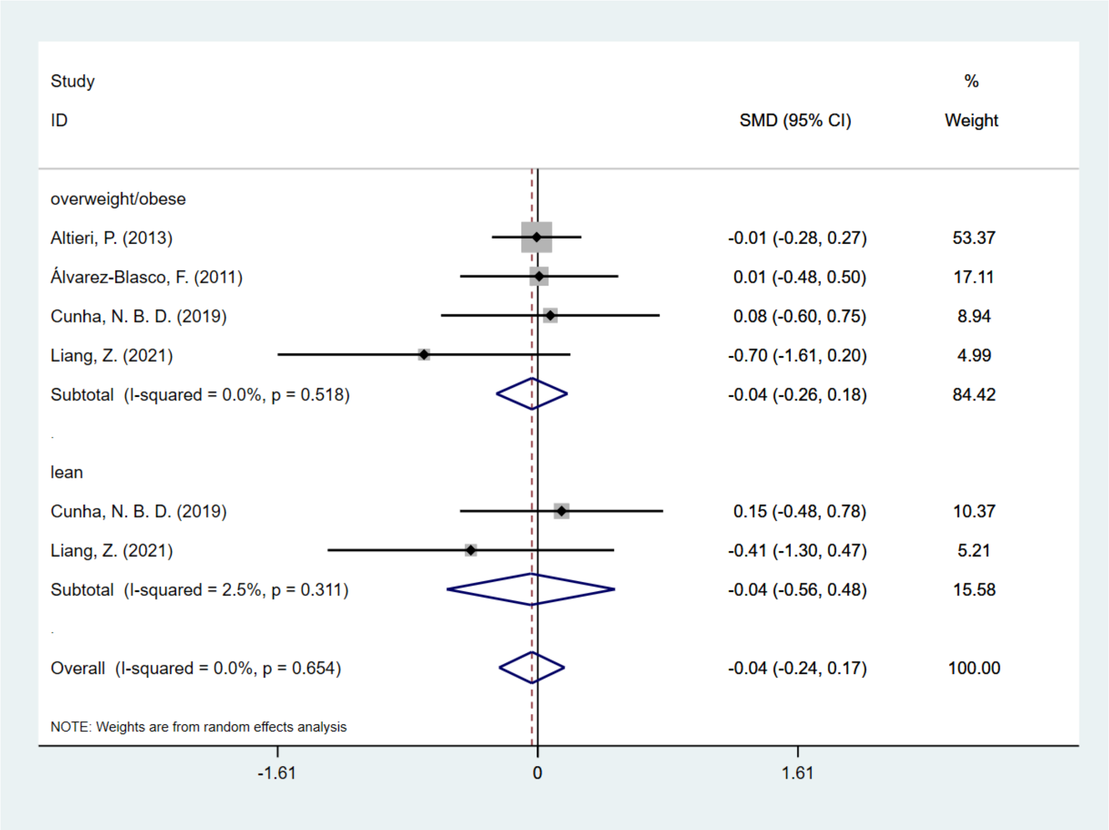

Figure S2. Meta-analysis of total energy intake by BMI classification [38–40,42].

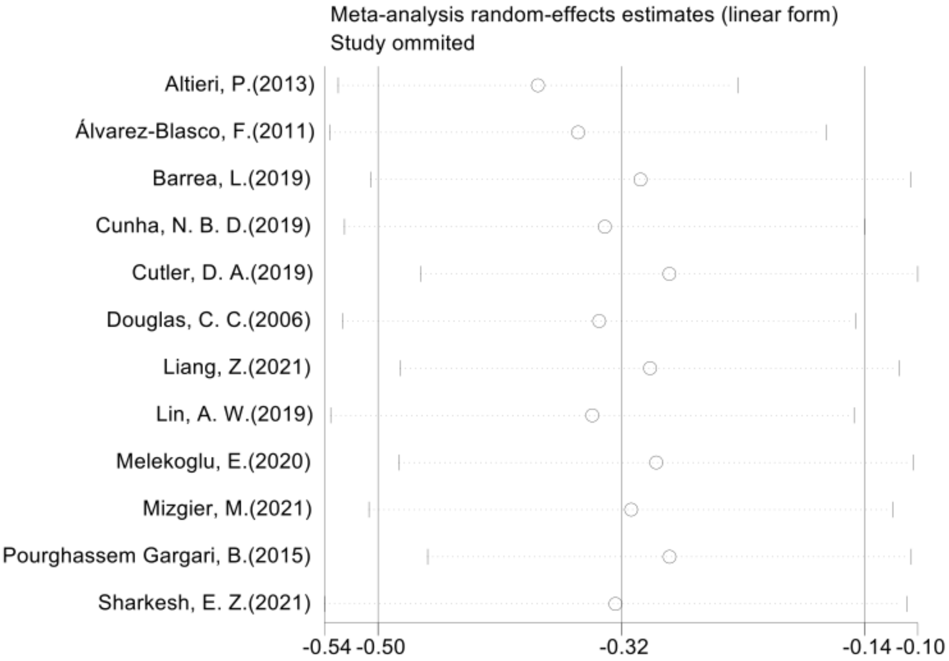

Figure S3. Influence analysis assessing the sensitivity of the pooling result on every single study [38–40 ,42–50].

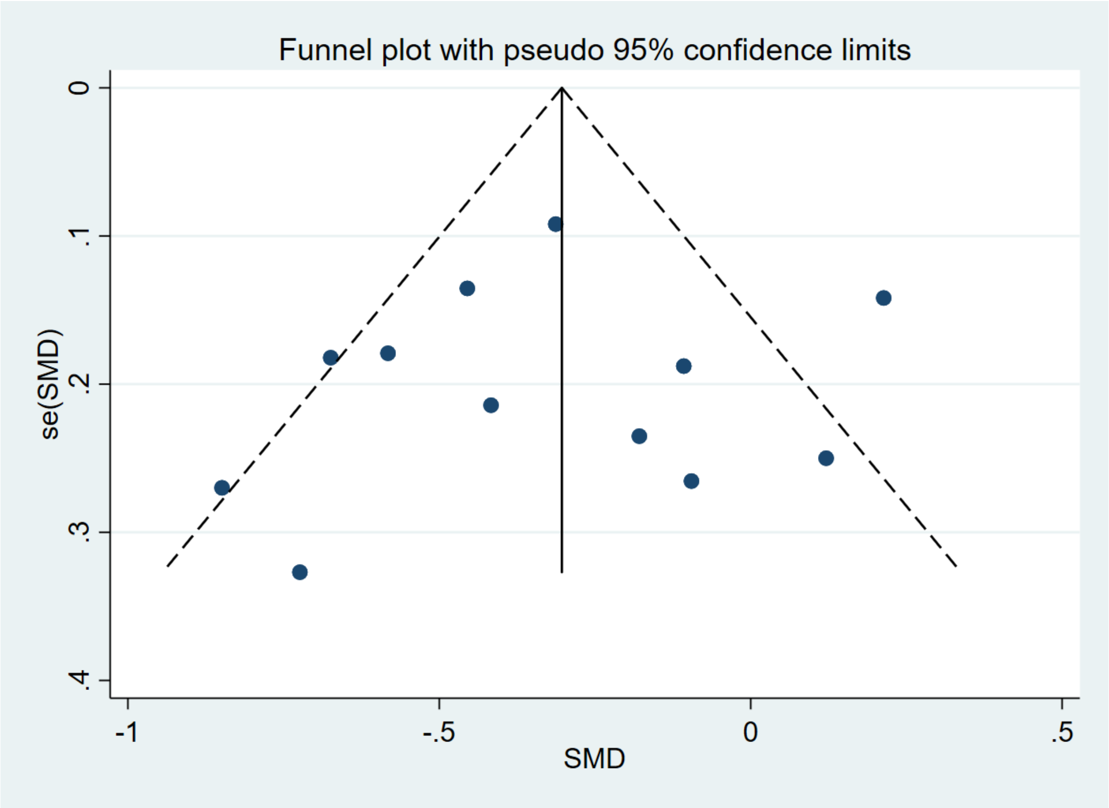

Figure S4. Funnel plot of the meta-analysis comparing dietary fiber intake on studies unadjusted for total energy intake.

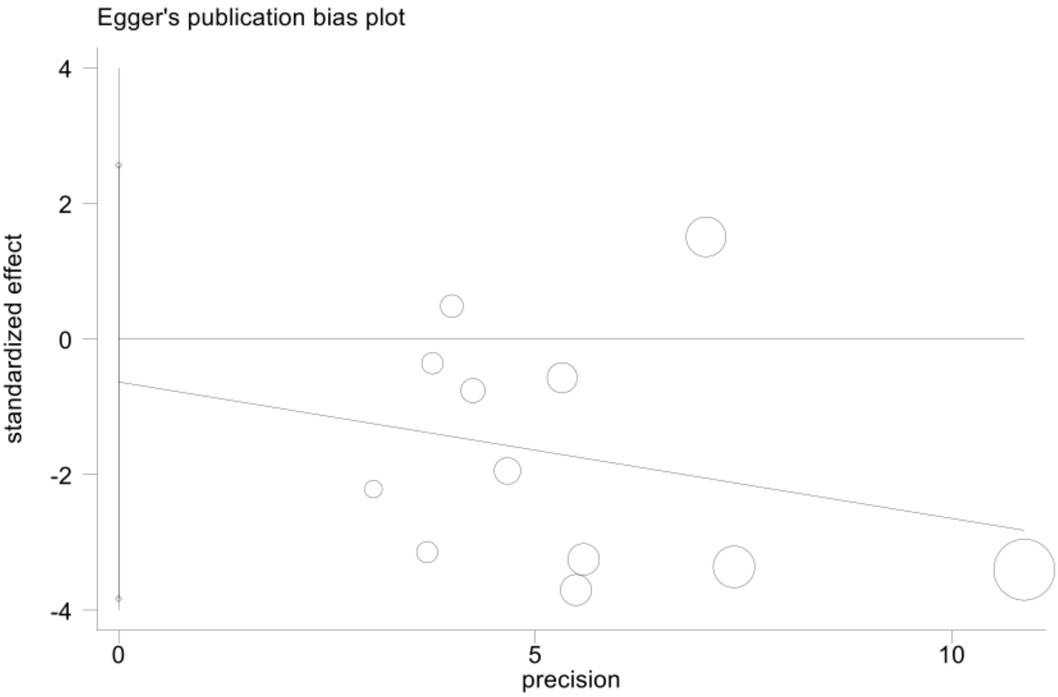

Figure S5. Egger’s publication bias plot. Graph symbol sized by study weights.

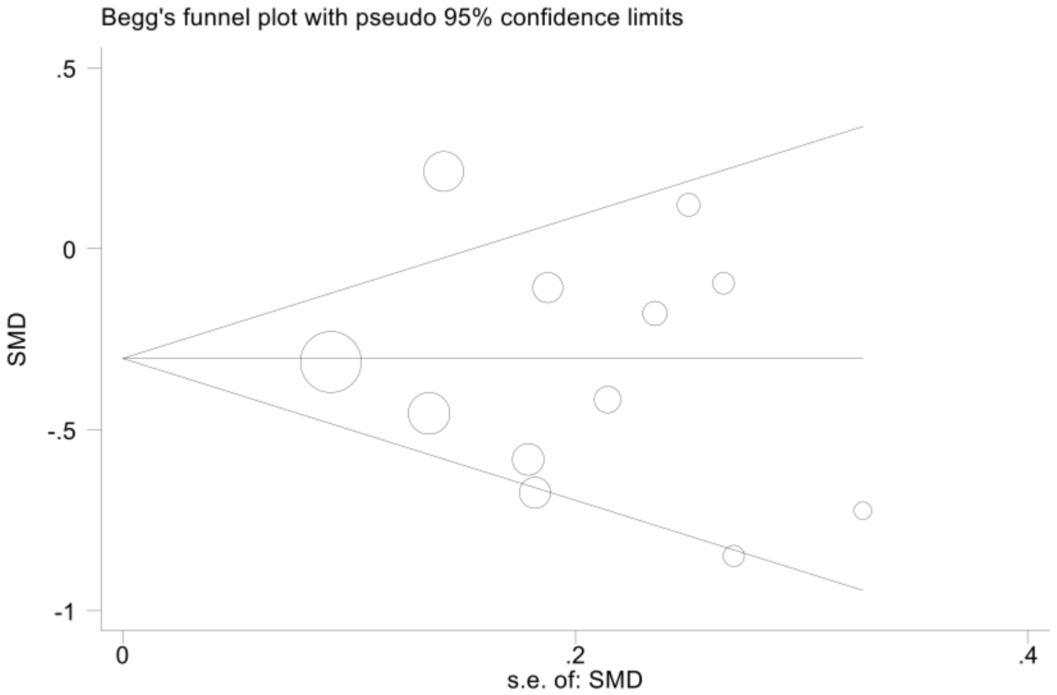

Figure S6. Begg’s publication bias plot. Graph symbol sized by study weights.

Table S1 Newcastle-Ottawa quality assessment of the included studies

Study	Selection	Comparability	Exposure/Outcome	Summation
Case-control/cross-sectional study				
Altieri, P.(2013) [38]	3	2	2	7
Álvarez-Blasco, F.(2011) [39]	3	-	2	5
Barrea, L.(2019) [48]	2	2	2	6
Cunha, N. B. D.(2019) [40]	4	2	2	8
Eslamian, G.(2017) [41]	3	1	2	6
Liang, Z.(2021) [42]	3	2	2	7
Lin, A. W.(2019) [43]	4	-	2	6
Melekoglu, E.(2020) [44]	2	1	2	5
Mizgier, M.(2021) [45]	3	1	2	6
Pourghassem Gargari, B.(2015) [46]	3	1	2	6
Sharkesh, E. Z.(2021) [47]	3	-	2	5
Cohort study				
Cutler, D. A.(2019) [49]	2	-	2	4
Douglas, C. C.(2006) [50]	2	2	2	6

- scoring no point in the corresponding category.
